# Re-exploration for bleeding after cardiac surgery: revaluation of urgency and factors promoting low rate

**DOI:** 10.1186/s13019-021-01545-4

**Published:** 2021-06-07

**Authors:** Ahmed Abdelrahman Elassal, Khalid Ebrahim Al-Ebrahim, Ragab Shehata Debis, Ehab Sobhy Ragab, Mazen Shamsaldeen Faden, Mazin Adel Fatani, Amr Ragab Allam, Ahmed Hasan Abdulla, Auhood Mohammednoor Bukhary, Nada Ahmed Noaman, Osama Saber Eldib

**Affiliations:** 1 Department of Surgery, Cardiac Surgery Unit, King Abdulaziz University, Jeddah, 21589 Saudi Arabia; 2 Cardiothoracic Surgery Department, Zagazig University, Zagazig, Egypt; 3 Department of Anesthesia and Critical Care, King Abdulaziz University, Jeddah, Saudi Arabia; 4 Department of Surgery, Umm Al-Qura University, Makkah, Saudi Arabia; 5 Department of Cardiac Surgery, Naser Institute of Research and Treatment, Cairo, Egypt; 6 Cardiothoracic Surgery Department, Alahrar Hospital, Zagazig, Egypt

**Keywords:** Bleeding, Cardiac surgery, Re-exploration

## Abstract

**Background:**

Re-exploration of bleeding after cardiac surgery is associated with significant morbidity and mortality. Perioperative blood loss and rate of re-exploration are variable among centers and surgeons.

**Objective:**

To present our experience of low rate of re-exploration based on adopting checklist for hemostasis and algorithm for management.

**Methods:**

Retrospective analysis of medical records was conducted for 565 adult patients who underwent surgical treatment of congenital and acquired heart disease and were complicated by postoperative bleeding from Feb 2006 to May 2019. Demographics of patients, operative characteristics, perioperative risk factors, blood loss, requirements of blood transfusion, morbidity and mortality were recorded. Logistic regression was used to identify predictors of re-exploration and determinants of adverse outcome.

**Results:**

Thirteen patients (1.14%) were reexplored for bleeding. An identifiable source of bleeding was found in 11 (84.6%) patients. Risk factors for re-exploration were high body mass index, high Euro SCORE, operative priority (urgent/emergent), elevated serum creatinine and low platelets count. Re-exploration was significantly associated with increased requirements of blood transfusion, adverse effects on cardiorespiratory state (low ejection fraction, increased s. lactate, and prolonged period of mechanical ventilation), longer intensive care unit stay, hospital stay, increased incidence of SWI, and higher mortality (15.4% versus 2.53% for non-reexplored patients). We managed 285 patients with severe or massive bleeding conservatively by hemostatic agents according to our protocol with no added risk of morbidity or mortality.

**Conclusion:**

Low rate of re-exploration for bleeding can be achieved by strict preoperative preparation, intraoperative checklist for hemostasis implemented by senior surgeons and adopting an algorithm for management.

## Introduction

Bleeding after cardiac surgery is a well-known serious complication increasing the rate of reexploration, requirement of blood transfusion, length of hospital stay and cost. It has an adverse impact on morbidity and mortality [1]. Reexploration for bleeding is associated with prolonged hospital stay and increased complications e.g. sternal wound infection (SWI), renal impairment, and postoperative arrhythmias [2]. Every effort should be exerted to avoid postoperative bleeding and to manage it properly if happens [3]. Variability of perioperative blood loss and rate of reexploration among centers and surgeons were reported (Fig. 5). Dixon B et al. reported that 798 surgical units in the United States found a range from 8 to 93% of patients requiring a blood transfusion. They confirmed that this variation could not be related to patient or other hospital factors and suggested that variations in surgeons’ hemostatic practices may be an important factor contributing to the extent of bleeding complications and the rate of re-exploration [4]. We present our experience of objectively low rate of re-exploration for bleeding after cardiac surgery. We introduce our strategy of management based on own checklist for hemostasis and roadmap for decision making.

## Patients and methods

**Fig. 1:**
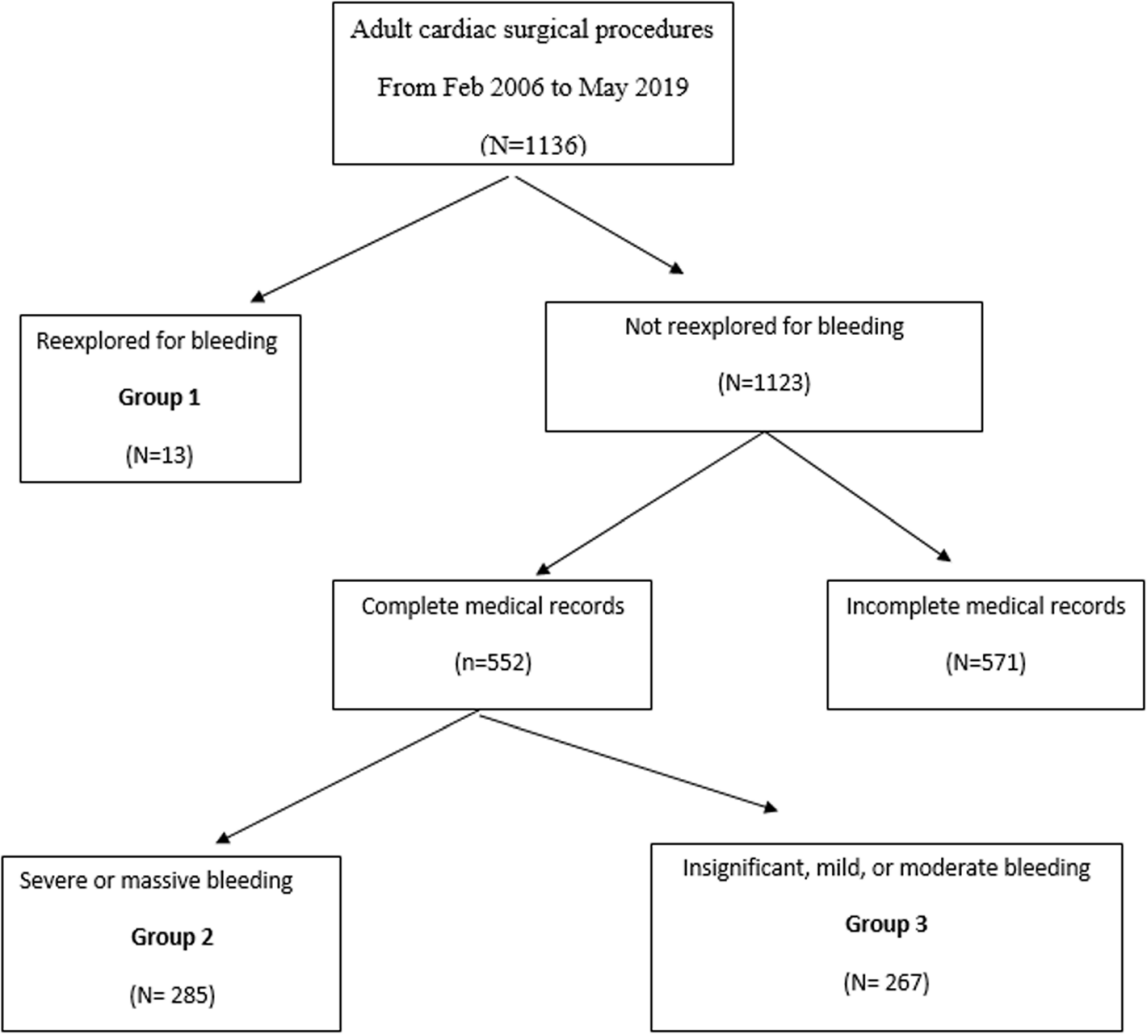
Study flow chart

From Feb 2006 to May 2019, we designed a retrospective observational analytic study for 565 adult patients underwent surgical treatment of congenital and acquired heart disease and were complicated by postoperative bleeding. The total recorded number of adult cardiac surgery cases in this period was 1136. The study has been approved by Ethics Committee (EC). The consent of patients was obtained. Study flow chart is shown in Fig. 1.

### Definitions

1. Operative priority

- Emergent: done immediately or within 24 h of diagnosis
- urgent: operations done during the same hospital admission

2. surgical bleeding: localized source is identified
3. Prolonged ventilatory support: > 24 h.
4. Re-exploration for bleeding: repeat surgery performed for bleeding or tamponade from time of ICU admission postoperatively till time of hospital discharge.
5. Mortality: hospital mortality at any time after index operation and before discharge

Demographics of patients, operative characteristics, perioperative risk factors, blood loss, requirements of blood transfusion, morbidity and mortality were recorded.

Patients were categorized into 3 groups according to universal definition of perioperative bleeding by Dyke et al. [3]:

- Group 1: patients explored for massive bleeding (blood loss within 12 h > 2000 ml).
- Group 2: patients with severe and massive bleeding managed conservatively
- Group 3: patients with insignificant, mild or moderate bleeding managed conservatively.

**Fig. 2:**
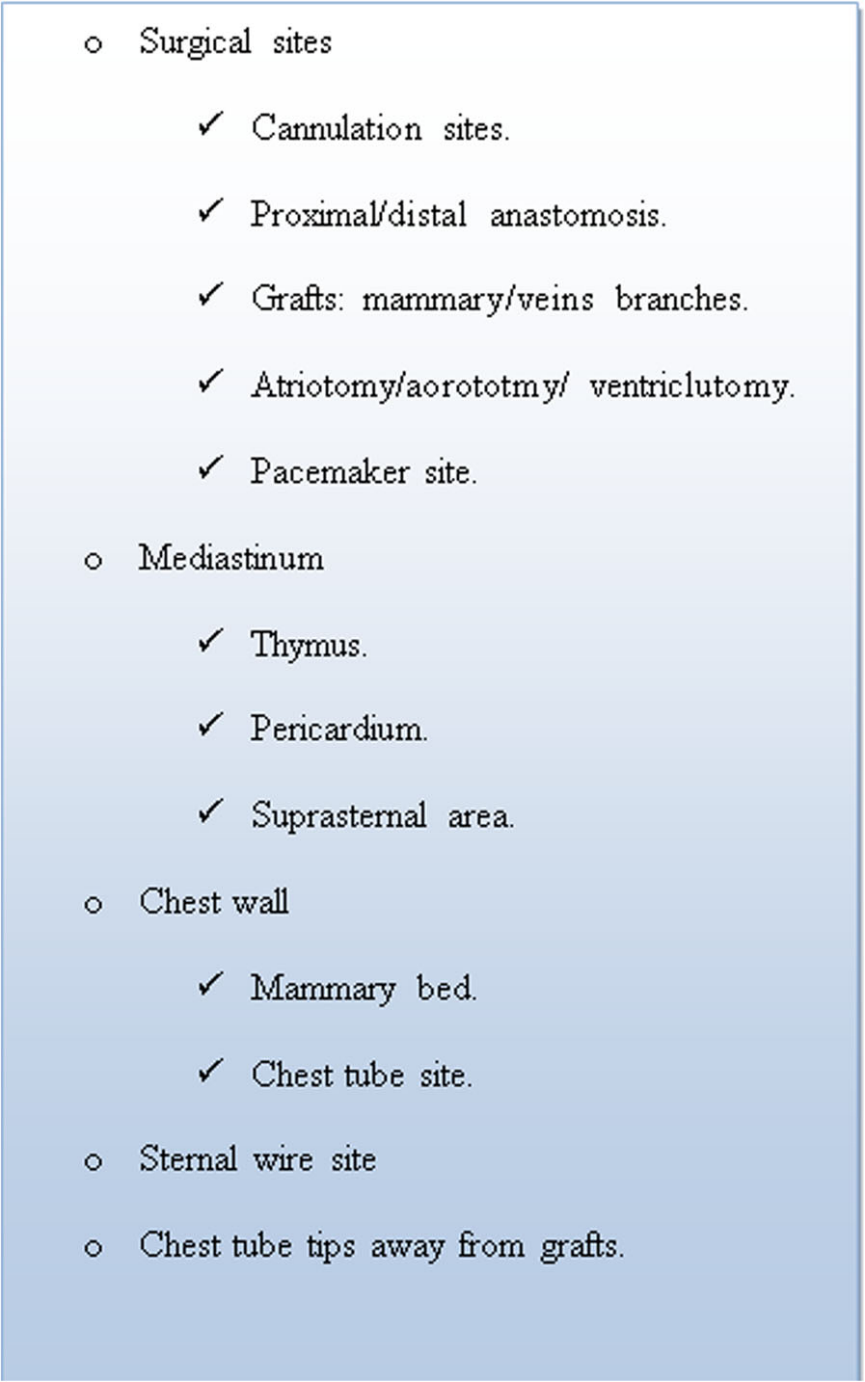
Authors᾿ Checklist for intraoperative hemostasis in cardiac surgery

Regarding preoperative medications, we stop warfarin 3 to 4 days and clopidogrel 5 days before operation while aspirin is continued till the time of surgery. All cases were conducted to conventional cardiopulmonary bypass (CPB) with full heparinization (loading dose of heparin 3 mg/kg and activated clotting time (ACT) is maintained > 480 s) and cold blood cardioplegia through full median sternotomy. Heparin is reversed with protamine sulphate on 1: 1 basis and extra dose of 25 mg is given till basal ACT is reached. All residual blood in reservoir was given to the patients. We use cell saver for patients with disseminated intravascular coagulopathy (DIC) and excessive intraoperative bleeding. Chest is left open in such circumstances. Before sternal closure and after completion of primary surgery, we applied the following planned perioperative checklist for meticulous stepwise hemostasis (Fig. 2). Closure was carried out by a fixed surgical team for all patients.

### Workup

During early postoperative care in the ICU or the cardiac surgery ward, periodic hematological test, coagulation profile, ACT and chest x ray were routine. Echocardiogram was done in suspected cardiac tamponade. The volume of blood loss was measured from all chest drains from the time of chest closure.

### Management of bleeding

**Fig. 3:**
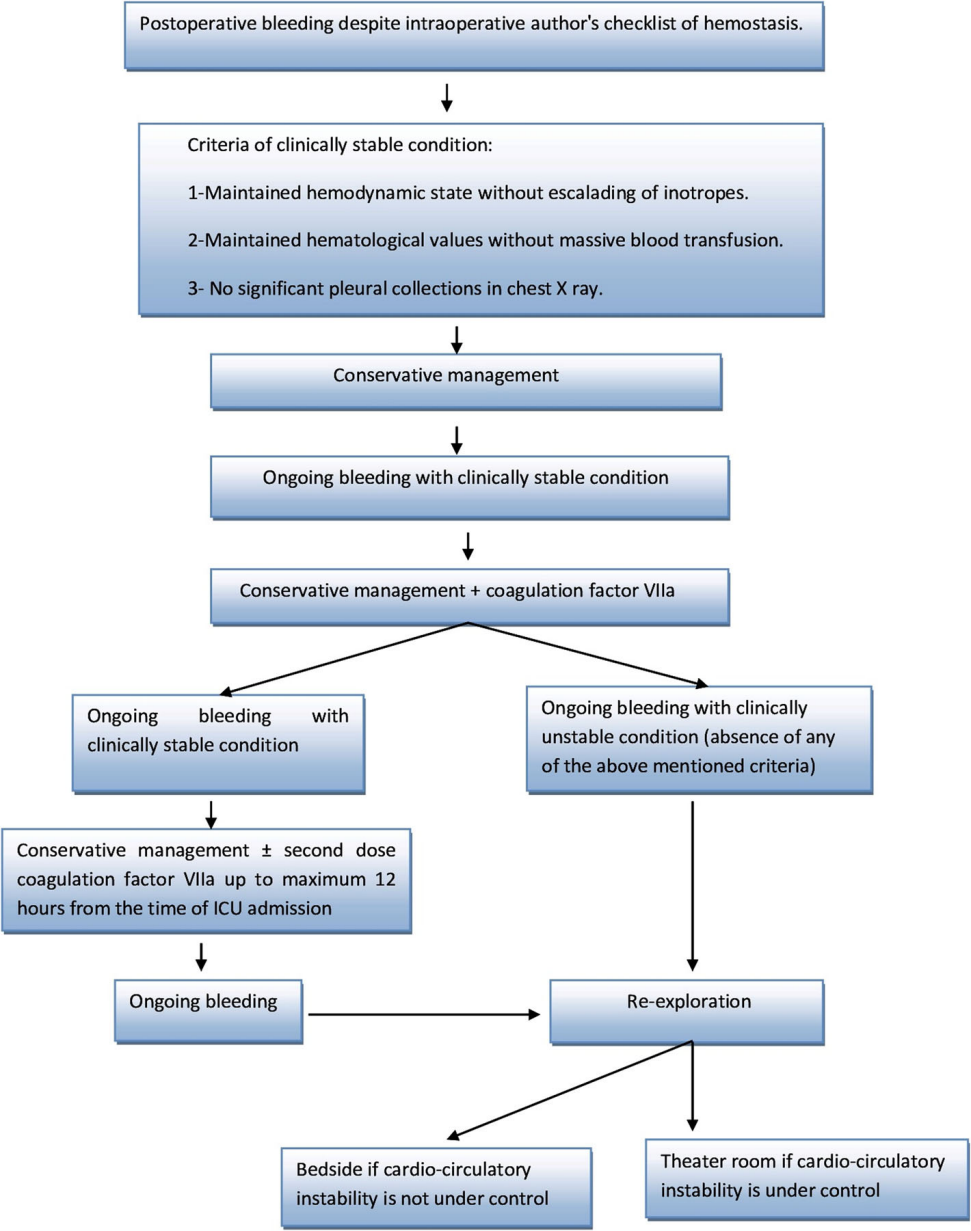
Authors᾿ roadmap of management of postoperative bleeding after cardiac surgery

Our roadmap for management of postoperative bleeding is shown in Fig. 3. Except for hemodynamically unstable patients, we follow conservative management in the form of transfusion of fresh blood products, factor VII, and tranexmic acid (1–2 g). We follow liberal strategy for blood transfusion. Packed RBCs are given if Hb is less than 8 g/dL with mixed venous saturation > 60% and if Hb is < 10 g/dL with mixed venous saturation < 60%. During the first 48 h after primary surgery, no definite time to switch from conservation to exploration as long as hemodynamics and hematological tests were controlled.

Re-sternotomy is carried out in OR except for patients with tamponade or cardiac arrest where it is done emergently bedside in ICU. The decision of reexploration is a teamwork decision involving cardiac surgery and ICU physicians and finally approved by the operating surgeon.

### Follow up

Period of follow up for all patients was during hospitalization.

### Statistical analysis

Statistical analysis was done using SPSS software version 27 **(IBM, 2020).** Shapiro–Wilk test was used to determine the distribution characteristics of variables and variance homogeneity. Normally distributed data were described using mean and SD, while non-normally distributed data were described using median and interquartile range (IQR). Qualitative data was presented as frequencies and proportions. Chi-square test (χ^2^) was used to test differences for categorical variables.

One-way ANOVA test (F) was used to test differences when more than two independent groups were present and variances were equal, while Kruskal-Wallis test (KW) was used when equal variances were not present. Binary logistic regression analysis was used to identify risk factors for re-exploration and determinants of adverse outcome. Variables were tested using Wald statistic. Odds ratio and 95% confidence interval were calculated to estimate the risk. A *P*-value of <0.05 was accepted as statistically significant.

## Results

### Prevalence and trends

**Fig. 4:**
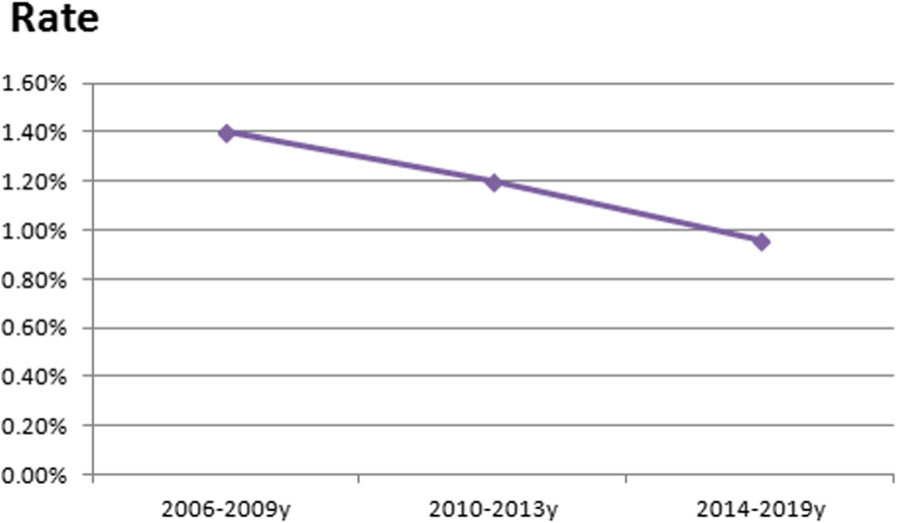
Time trend of percentage of rate of re-exploration for bleeding after cardiac surgery from the year 2006 to the year 2019 in authors᾿ institute. Rate: percentage of rate of re-exploration

**Fig. 5:**
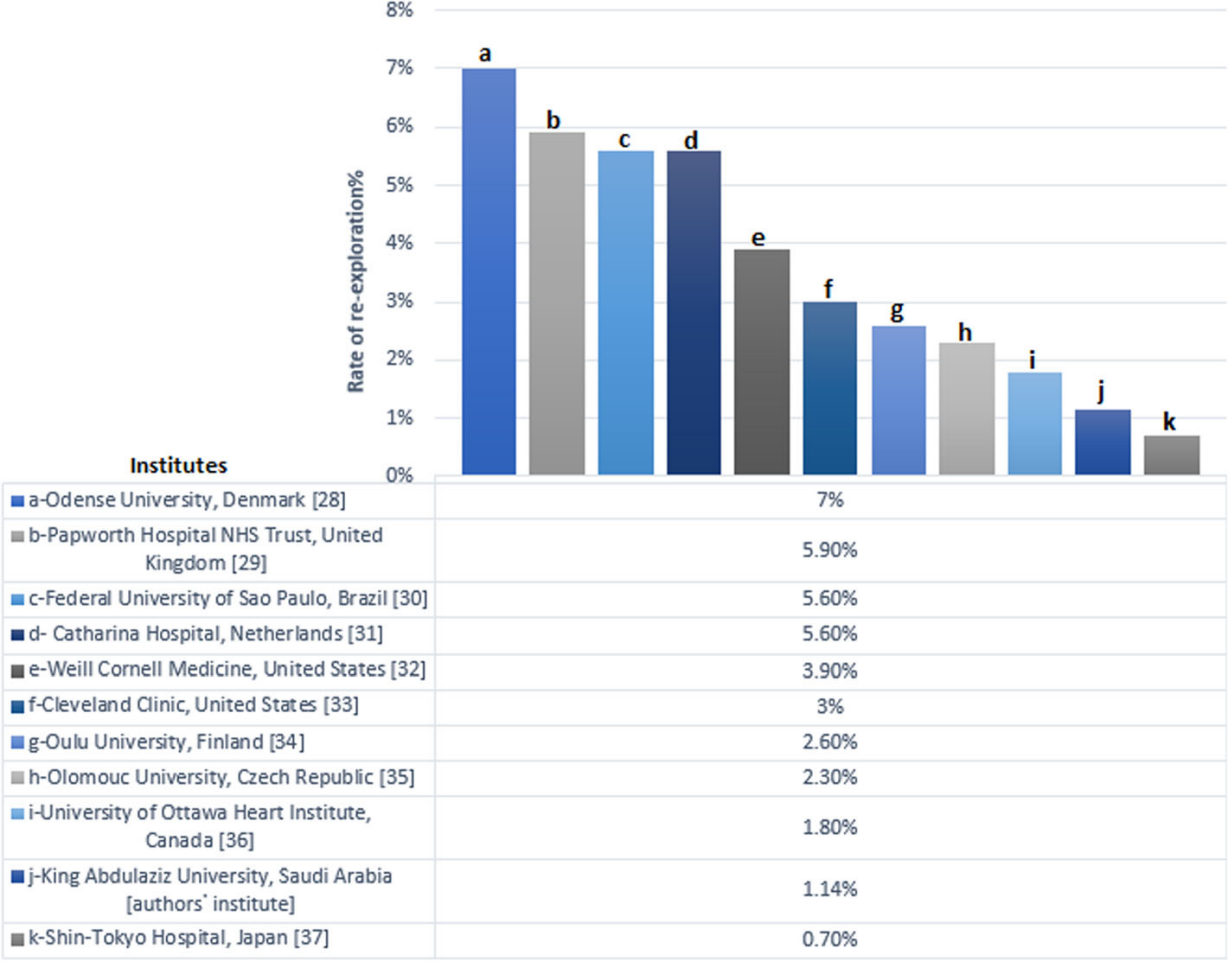
Comparing the percentages of rate of re-exploration for bleeding after cardiac surgery in authors᾿ institute versus other institutes

Thirteen patients (1.14%) were reexplored for bleeding in the period between 2006 and 2019. Declined trend was noted with prevalence falling during the last 7 years (Fig. 4). Our incidence is lower than many well- known institutes around the world (Fig. 5).

### Perioperative characteristics

Table 1 represents the preoperative characteristics of the 3 groups. No significant differences were found regarding age, sex, BSA, preoperative EF, cardiac risk factors (DM, hypertension, and smoking), preoperative medications, and basal ACT. Group 1 (reexploration group) had higher BMI, NYHA class, Euro SCORE, serum creatinine, and higher percentage of patients operated on urgent and emergent basis. Almost one third of this group had chronic lung diseases. Patients in group 1 had the lowest platelets count. Operative variables are shown in Table 2. No significant differences were found regarding type of surgery, bypass time, clamping time, lowest HCT level, lowest temperature during bypass, and ACT after reversal of heparin. Patients in group 1 showed increased requirement for intraoperative blood transfusion and mechanical assist devices. Surgical source of bleeding was found in 11 (84.6%) of reexplored patients (Table 3).

**Table 1:** Preoperative patient characteristics

Variables	Group 1 (***n*** = 13)	Group 2 (***n*** = 285)	Group 3 (***n*** = 267)	P
Age, median (IQR), y	51.0 (40.0–57.5)	55.0 (47.0–62.0)	54.0 (44.0–61.0)	0.6
Sex, n (%)				
Male	10 (76.9%)	228 (80.0%)	194 (72.7%)	0.3
Female	3 (23.1%)	57 (20.0%)	73 (27.3%)	
BSA, median (IQR), m^2^	1.8 (1.7–2.0)	1.8 (1.7–1.9)	1.8 (1.6–1.9)	0.5
BMI, n (%)				
Under weight	1 (7.7%)	93 (32.6%)	76 (28.5%)	**<0.001**
Average	6 (46.2%)	189 (66.3%)	189 (70.8%)	
Obese	6 (46.2%)	3 (1.1%)	2 (0.7%)	
NYHA functional class, n (%)				
I	0 (0.0%)	24 (8.4%)	73 (27.3%)	**<0.001**
II	7 (53.8%)	216 (75.8%)	138 (51.7%)	
III	5 (38.5%)	44 (15.4%)	49 (18.4%)	
IV	1 (7.7%)	1 (0.4%)	7 (2.6%)	
Canadian score, n (%)				
0	0 (0.0%)	67 (23.5%)	40 (15.0%)	**0.003**
1	4 (30.8%)	111 (38.9%)	99 (37.2%)	
2	8 (61.5%)	55 (19.3%)	70 (26.3%)	
3	1 (7.7%)	51 (17.9%)	52 (19.5%)	
4	0 (0.0%)	1 (0.4%)	5 (1.9%)	
EuroSCORE, median (IQR)	1.3 1.25–1.6	0.97 0.9–1.3	1.0 0.9–1.3	**<0.001**
Ejection fraction, n (%)				
> 50%	5 (38.5%)	130 (45.6%)	140 (52.4%)	0.2
30–50%	8 (61.5%)	144 (50.5%)	122 (45.7%)	
< 30%	0 (0.0%)	11 (3.9%)	5 (1.9%)	
Preoperative shock, n (%)	1 (7.7%)	8 (2.7%)	8 (2.9%)	0.6
Atrial fibrillation, n (%)	1 (7.7%)	37 (12.3%)	39 (14.0%)	0.7
Operative priority, n (%)				
Elective	9 (69.2%)	268 (94.0%)	237 (88.8%)	**<0.001**
Urgent	2 (15.4%)	14 (4.9%)	23 (8.6%)	
Emergent	2 (15.4%)	3 (1.1%)	7 (2.6%)	
Hypertension, n (%)	9 (69.2%)	139 (48.8%)	132 (49.4%)	0.3
Diabetes mellitus, n (%)	8 (61.5%)	141 (49.5%)	144 (53.9%)	0.4
Smoking, n (%)	5 (38.5%)	107 (37.6%)	94 (35.2%)	0.9
Chronic lung disease, n (%)	5 (38.5%)	37 (13.0%)	16 (6.0%)	**<0.001**
Stroke, n (%)	0 (0.0%)	2 (0.7%)	5 (1.9%)	0.4
PVD, n (%)	2 (15.4%)	10 (3.5%)	8 (3.0%)	0.06
Anemia, n (%)	3 (23.1%)	122 (43.1%)	102 (38.2%)	0.2
Preoperative medications, n (%)				
Aspirin,	7 (53.8%)	168 (58.9%)	134 (50.2%)	0.1
Plavix	5 (38.5%)	69 (24.2%)	67 (25.1%)	
Warfarin	1 (7.7%)	47 (16.9%)	66 (24.7%)	
Platelets count, median (IQR), ×10^9^/L	215.5 (179.5–238.5)	245.3 (225.0–338.8)	235.7 (214.5–301.5)	**<0.001**
Serum creatinine, median (IQR), μmol/L	114.9 (86.0–156.0)	88.0 (74.0–99.0)	89.9 (75.0–104.0)	**0.02**
Based ACT, median (IQR), sec	121.6 (112.0–132.5)	128.2 (118.3–139.0)	127.8 (116.0–137.0)	0.2

**Table 2:** Operative characteristics

Variables	Group 1 (***n*** = 13)	Group 2 (***n*** = 285)	Group 3 (***n*** = 267)	P
Type of surgery, n (%):				
CABG	9 (69.2%)	224 (78.6%)	187 (70.0%)	0.2
mitral valve replacement	2 (15.4%)	39 (13.7%)	42 (15.8%)	
combined operation	0 (0.0%)	2 (0.7%)	6 (2.2%)	
Others	2 (15.4%)	20 (7.0%)	32 (12.0%)	
CBP time, median (IQR), min	105.5 (78.5–140.8)	108.5 (84.8–139.0)	109.5 (84.0–150.8)	0.3
Clamping time, median (IQR), min	61.5 (45.5–99.0)	65.0 (49.8–84.3)	64.0 (50.0–89.0)	0.4
Mechanical assist devices (ECMO, IAB), n (%):	2 (15.4%)	9 (3.2%)	5 (1.9%)	**0.01**
Lowest HCT, Mean ± SD	17.8 ± 3.4	17.5 ± 3.0	17.6 ± 3.2	0.8
ACT after protamine, Mean ± SD, sec	131.5 ± 12.6	132.3 ± 29.2	130.7 ± 30.6	0.8
Lowest temperature during CPB, Mean ± SD	28.0 ± 3.2	29.2 ± 2.4	29.2 ± 2.6	0.2
Drainage, median (IQR), ml	1735.0 (1352.5–2201.3)	800.0 (650.0–1000.0)	690.0 (460.0–860.0)	**<0.001**
Intraoperative transfusion of Packed RBCs, median (IQR), units	2.0 (2.0–3.0)	2.0 (1.0–3.0)	2.0 (1.0–2.0)	**<0.001**

**Table 3:** Characteristics of reexplored patients

Cause of reexploration	Tamponade	5
	Cardiac arrest	2
	Massive bleeding	6
Time from primary operation to reexploration, Median (IQR), hours	23 (9–192)	
Site of bleeding	Proximal anastomosis	2
	Distal anastomosis	2
	Atriotomy	2
	LIMA	1
	Chest wall (mammary bed)	2
	RV	1
	IVC	1
	Unidentified (diffuse bleeding)	2
Outcome	Survived	11
	Died	2

### Predictors of reexploration

After binary logistic regression analysis of preoperative risk factors BMI, Euro SCORE, operative priority (urgent/emergent), and elevated serum creatinine and low platelets count were significantly associated with increased risk of reexploration (Table 4).

**Table 4:** Binary logistic regression analysis of preoperative risk factors for re-exploration for bleeding

Variable	SE	Wald	Odds Ratio (95% Confidence interval)	P
BMI	0.14	29.7	2.1 (1.6–2.8)	**0.003**
NYHA functional class	0.5	3.2	2.3 (0.9–5.7)	0.07
EuroSCORE	0.7	6.4	5.5 (1.5–20.6)	**0.006**
Operative priority (urgent/emergent)	0.8	11.6	2.9 (1.6–14.8)	**0.02**
Chronic lung diseases	0.9	1.9	3.5 (0.6–20.8)	0.1
Platelets count	0.01	5.6	0.98 (0.96–0.99)	**0.01**
Serum creatinine	0.14	29.7	2.1 (1.6–2.8)	**0.003**

### Impact of reexploration on outcome

Patients reexplored for bleeding had greater postoperative blood loss from chest drains (median 1735 mL, IQR 1352.5–2201.3 mL) and increased requirement for postoperative transfusion of packed RBCs, FFP, and platelets. Adverse effects on cardiorespiratory state (low EF, increased s. lactate, and prolonged period of MV) were more evident in group 1. In addition, reexploration for bleeding was significantly associated with longer ICU stay, hospital stay, and increased incidence of SWI. Higher mortality rate was found in patients who underwent reexploration (15.4%) versus 2.53% for patients who did not (Table 5).

**Table 5:** Postoperative Characteristics and outcome

Variables	Group 1 (***n*** = 13)	Group 2 (***n*** = 285)	Group 3 (***n*** = 267)	P
Postoperative transfusion of Packed RBCs, median (IQR), unit	11.0 (5.0–13.0)	2.0 (2.0–4.0)	1.0 (0.0–2.0)	**<0.001**
Fresh frozen plasma, median (IQR), unit	12.0 (9.5–15.0)	4.0 (4.0–6.0)	3.0 (2.0–4.0)	**<0.001**
Platelets, median (IQR), unit	9.1 (6.0–12.0)	6.2 (4.0–6.0)	3.3 (0.0–2.0)	**<0.001**
Serum lactate, median (IQR), unit mmol/L	3.0 (2.0–3.0)	2.0 (1.0–2.0)	2.0 (1.0–2.0)	**<0.001**
ACT, median (IQR), sec	120.0 (117.3–143.8)	117.0 (110.0–122.3)	112.0 (101.5–120.0)	**<0.001**
PT, median (IQR), sec	12.0 (10.3–14.3)	12.0 (11.0–12.2)	12.0 (11.0–18.0)	**<0.001**
Postoperative EF	37.5 (31.3–45.0)	40.0 (35.0–50.0)	45.0 (40.0–55.0)	**<0.001**
Serum creatinine, median (IQR), μmol/L	116.0 (83.5–191.0)	99.0 (85.0–120.0)	91.0 (84.0–117.0)	0.09
Ventilator hours, median (IQR), hrs	35.5 (24.5–197.3)	14.0 (8.0–20.0)	14.0 (8.0–24.0)	**<0.001**
ICU stay, median (IQR), days	3.5 (2.3–6.8)	2.0 (2.0–3.3)	3.0(2.0–24.0)	**<0.001**
Hospital stay, median (IQR), days	13.0 (11.5–17.5)	7.0 (7.0–8.0)	7.0 (6.0–8.0)	**<0.001**
Deep sternal wound infection, n (%)	2 (15.4%)	12 (4.2%)	6 (2.2%)	**0.03**
Mortality, n (%)	2 (15.4%)	13 (4.6%)	1 (0.4%)	**<0.001**

### Risk factors of adverse outcome

Binary logistic regression analysis of operative and postoperative determinants had shown that transfusion of packed RBCs, FFP, increased serum lactate, prolonged MV, and long ICU and hospital were associated with adverse outcome (Table 6).

**Table 6:** Binary logistic regression analysis of operative and postoperative determinants of adverse outcome

Variable	SE	Wald	Odds Ratio (95% Confidence interval)	P
Requirement for Packed RBCs transfusion	0.3	10.7	2.3 (1.4–3.8)	**0.001**
Requirement for Fresh frozen plasma	0.3	8.2	0.4 (0.2–0.7)	**0.004**
Serum lactate	0.3	11.1	2.9 (1.5–5.4)	**0.001**
Platelets	0.3	3.5	1.6 (1.0–2.7)	0.06
Serum creatinine	0.03	0.004	1.0 (0.9–1.0)	0.9
Ventilator hours	0.001	5.3	1.1 (1.1–1.1)	**0.01**
ICU stay	0.04	5.5	1.1 (1.0–1.2)	**0.01**
Hospital stay	0.01	3.8	0.98 (0.96–0.99)	**0.004**

## Discussion

Bleeding after cardiac surgery is a well-known complication. Colson PH et al. reported that the incidental rate of active bleeding varied between centers (0 to 16%) but was independent of in-center cardiac surgical experience [5]. They claimed that variability is due to the arguments about the definitions of active bleeding and what is the significant bleeding or the insignificant bleeding? [5]. Increase in chest tube drainage (> 200 ml/hr. in any single hour, > 2 ml/kg/hr. in 2 successive hours, or > 495 ml in first 24 h) was associated with higher morbidity and mortality [6]. Overall incidence of re-exploration for bleeding ranges from 2.3 to 6% [1, 7–10].

As regards type of operation, reported incidence was 4.5% for CABG, 5.5% for single valve, 9.6% for combined and 15% for emergency operations [8]. Our overall rate of reexploration for bleeding was 1.14% with a declined trend from 1.4% (2006–2009) to 0.96% (2014–2019). We believe that adopting a checklist for hemostasis and algorithm for management could achieve a declined trend of re-exploration. The reported risk factors for reexploration for bleeding include emergency state, redo, low BSA, high Euro SCORE, dual antiplatelets less than 5 days before operation, on pump surgery, combined valve and CABG, long bypass and clamping times, and lowest hematocrit (24%). Debate exists about increasing age, preoperative renal dysfunction, and LV grade as risk factors for reexploration [5, 8, 10, 11]. Risk factors identified in our series were high BMI, high Euro SCORE, renal impairment, low preoperative platelet count, and operations performed on urgent or emergent basis. Poor outcome for reexplored patients was reported even after the first 3 months postoperatively [8]. Perioperative mortality increases 3.5 to 4.5-fold greater than patients not re-explored (12% versus 2.8%). This could be attributed to more blood transfusion, systemic hypotension and multi organ failure in reexplored patients [1, 5]. In a large study conducted by Vivacqua et al. on 18,752 patients, reoperation rate was 3% (2.3–4%) and mortality for reoperation was 8.5 vs 1% [11].. Mehta et al. evaluated 528,686 CABG patients operated at more than 800 hospitals in the Society of Thoracic Surgeons National Cardiac Database (2004 to 2007). Incidence of reoperation was 2.4% and mortality rate of re-explored patients was 9.1 vs 2% for non-reexplored [9]. Our results confirm this finding. Our re-explored group had higher mortality rate when compared to non reexplored patients (15.4% vs 2.53%). Reexploration was independent risk factors for morbidities including: renal failure, stroke, ARDS, sepsis and atrial arrhythmias, prolonged MV and mechanical circulatory support [11, 12]. Re-explored patients in our study had higher incidence of DSWI, longer periods of MV, ICU stay, and hospital stay and greater requirements for blood transfusion. Risk for poor outcome was related to both reoperation and transfusion requirements independently [9]. Transfusion of even 1 or 2 units of packed RBCs after CABG is associated with increased morbidity and mortality [13].

Timing of re-exploration greatly affects outcome. Delayed reexploration (> 12 h) is associated with worse outcome and an increased mortality up to 37.5% [5, 14]. Karthik and colleagues recommended early reoperation since surgical bleeding was found in 82% of re-exploration for bleeding [15]. Nine of our patients were reexplored after 12 h. Our policy delays reexploration provided hemodynamic stability is maintained and replacement by proper blood products is adequate. Low number of our reexplored patients could not allow comparison between those who were reexplored early and late. Decision to reexplore for bleeding was based mainly on flow of bleeding from chest tube or the volume of blood transfusion required for replacing blood loss [7]. Colson et al. in a large multicentre trial (29 centers, 4904 patients) defined active bleeding on a dynamic base that was blood loss > 1.5 ml/kg /hr. for 6 consecutive hours within the first 24 h [12]. We applied this definition in our algorithm but we start active management within the first 3 h. Causes of bleeding might be surgical (70%), coagulopathy (12%), or combined (9.5%). Common sites of bleedings include sternum, mammary bed, coronary anastomosis site [10]. Factors contributing to non-surgical bleeding include: hypothermia, hemodilution, consumption of coagulation factors and platelets by CPB circuit, platelet dysfunction, systemic inflammatory response of ECC, activation of fibrinolytic system, effect of preoperative medications (e.g. clopidogrel, aspirin, and warfarin) [16]. Review of literature and metanalysis of 18 studies was performed by Biancari et al. including 51,497 adult patients who underwent cardiac surgery, re-exploration for bleeding or tamponade was performed in 2455 patients. Surgical sites of bleeding were identified in 65.7% of cases. We had found surgical cause of bleeding in 11/13 of our patients. We agree with Biancari et al. that meticulous surgical technique and systematic check of potential sites of bleeding before sternal closure could prevent this complication. Patients reexplored for identifiable source of bleeding had lower mortality rate than patients with diffuse bleeding [17]. Loor et al. introduced a checklist of intraoperative hemostasis with a resultant decrease of re exploration rate from 3.1 to 1.9% [18]. We had our own checklist and a fixed team of surgeons are responsible for closure. Our protocol necessitates liberal strategy of blood transfusion. Liberal strategy for blood transfusion is associated with decreased mortality and morbidity compared to restrictive [2]. Use of FFP is recommended by ASA in case of active bleeding associated with reduction in coagulation factor level [19]. NIH and ASA recommend platelet transfusion for active bleeding associated with thrombocytopenia (platelets < 50,000 μ L-^1^) or abnormal platelet function [20]. Hemostatic medications play a vital role in management of perioperative bleeding. Munoz et al. reported 46% decline in reoperation in time related trend with Northern New England Cardiovascular Disease Study Group (period from 95 to 97 vs 92–94) despite increase prevalence of risk factors. Increased use of antifibrinolytics was the reason [21]. Tranexamic acid use is associated with decreased incidence of allogenic blood transfusion and rate of re-exploration [22]. Indications of rFVIIa after cardiac surgery are life threatening bleeding (e.g intracranial, or > 500–1000 ml/hr), and bleeding not responding to usual hemostatic agents (1–2 U platelets, 4–8 U FFP, 10–20 U cryoprecipitate) [23]. According to universal definition of perioperative bleeding by Dyke et al. [4], patients in Group 2 (*n* = 285) were classified as having severe or massive bleeding. They were managed nonoperatively by hemostatic agents according to our protocol with no added risk of morbidity or mortality when compared to control group 1. This supports our theory that lower rate of re-exploration could be achieved. Planned re-exploration for bleeding in ICU for hemodynamically stable patients is safe without increased risk of infection [24]. When we decide for reexploration we prefer doing it in OR reserving re-sternotomy in ICU for unstable patients and those with cardiac arrest. Full equipment of OR including heart lung machine and IAB facilitate the job. Crash on site re-exploration was done in only three cases of massive surgical bleeding from anastomosis causing cardiac arrest. None of our patients needed delayed sternal closure. We agree with Crawford et al. reported that delayed sternal closure for intraoperative ongoing bleeding is a safe alternative to unplanned reexploration without additive risk of morbidity (including DSWI) or mortality [25]. Effective collaboration (multi-disciplinary teams) between cardiac surgeon, perfusionist, anesthetist, clinical pharmacist, and ICU physician is required to prevent and manage postoperative bleeding. Use of algorithm can decrease morbidity including allogenic blood transfusion [26]. We noticed that the incidence of bleeding and re-exploration in our cardiac unit is less than most of the international cardiac centres as showen in Fig. 5 [27–36]. We searched the reasons for this low incidence and found it to be multi factorial. These include strict preoperative preparation regarding coagulation profile, anticoagulation drugs, and antiplatelet drugs. Another logistic cause in our unit is the small cardiac surgery service that although it is challenging and the complication rate is higher than large centres, the complication of bleeding and re-exploration are more controllable due to more care and focusing by same senior surgeons for small number of patients [37]. In our unit standard checklist is implemented and followed by the same senior surgeons. Efficient blood bank and pharmacy are available to supply all required blood products and haemostatic drugs including antifibrinolytics and factor VII. Intraoperative haemostasis steps are applied by senior cardiac surgeons to assure adequate and almost perfect haemostasis using topical agents in addition to other standard steps. In all cases of bleeding in the intensive care unit, multidisciplinary decision is taken.

### Limitations

This is a retrospective observational study from single institute of modest volume of cases to focus on our experience of low rate of re-exploration for bleeding after cardiac surgery. As the topic of bleeding and hemostasis in cardiac surgery has wide arguments among those who prefer conservation and delay re-exploration versus those who decide to re-explore early, so analysis of non-hemorrhagic recorded cardiac surgery cases, data collection of large volume of cases from multiple centers, and prospective investigation of checklists, protocols and algorithms of bleeding and hemostasis, are important issues for producing more conclusive studies and determining more relevant clinical outcomes.

## Conclusion

Low rate of re-exploration for bleeding can be achieved by strict preoperative preparation, intraoperative checklist for hemostasis implemented by senior surgeons and adopting an algorithm for management.

## Data Availability

The datasets used and/or analysed during the current study are available from the corresponding author on reasonable request.

## Abbreviations

ACT: Activating Clotting Time
ARDS: Acute Respirtory Distress Syndrome
ASA: American Society of Anesthesiologists
BMI: Body Mass Index
CABG: Coronary Artery Bypass Grafting
DIC: Disseminated Intravascular Coagulopathy
EC: Ethical Committee
FFP: Fresh Frozen Plasma
IAB: Intra-aortic Balloon
ICU: Intensive Care Unit
IQR: Interquartile Range
KW: Kruskal-Wallis test
MV: Mechanical Ventilation
NIH: National Institute of Health
NYHA: New York Heart Association
PRBCs: Packed Red Blood Cells
SWI: Sternal Wound Infection
